# Differential signalling requirements for RIPK1-dependent pyroptosis in neutrophils and macrophages

**DOI:** 10.1038/s41419-024-06871-8

**Published:** 2024-07-04

**Authors:** See Jie Yow, Safwah Nasuha Rosli, Paul E. Hutchinson, Kaiwen W. Chen

**Affiliations:** 1 Immunology Translational Research Programme, Department of Microbiology and Immunology, Yong Loo Lin School of Medicine, National University of Singapore, Singapore, Singapore; 2 Immunology Programme, Life Sciences Institute, National University of Singapore, Singapore, Singapore

**Keywords:** Cell death and immune response, Infection

## Abstract

TLR4 and TNFR1 signalling promotes potent proinflammatory signal transduction events, thus, are often hijacked by pathogenic microorganisms. We recently reported that myeloid cells retaliate *Yersinia* blockade of TAK1/IKK signalling by triggering RIPK1-dependent caspase-8 activation that promotes downstream GSDMD and GSDME-mediated pyroptosis in macrophages and neutrophils respectively. However, the upstream signalling events for RIPK1 activation in these cells are not well defined. Here, we demonstrate that unlike in macrophages, RIPK1-driven pyroptosis and cytokine priming in neutrophils are driven through TNFR1 signalling, while TLR4-TRIF signalling is dispensable. Furthermore, we demonstrate that activation of RIPK1-dependent pyroptosis in neutrophils during *Yersinia* infection requires IFN-γ priming, which serves to induce surface TNFR1 expression and amplify soluble TNF secretion. In contrast, macrophages utilise both TNFR1 and TLR4-TRIF signalling to trigger cell death, but only require TRIF but not autocrine TNFR1 for cytokine production. Together, these data highlight the emerging theme of cell type-specific regulation in cell death and immune signalling in myeloid cells.

## Introduction

Myeloid cells such as macrophages, monocytes and neutrophils are sentinels of the innate immune system and express a variety of receptors to rapidly respond to microbial infection. For instance, Toll-like receptor 4 (TLR4) detects lipopolysaccharide (LPS) from Gram-negative bacteria, while tumour necrosis factor receptor 1 (TNFR1) senses the proinflammatory cytokine, TNF, that is often elevated during microbial infection. Activation of TNFR1 or TLR4 triggers the assembly of membrane-anchored multiprotein signalling platforms termed Complex I [1] or Myddosome [2], respectively, which initiate inflammation by triggering downstream signal transduction pathways that activate NF-κB and mitogen-activated protein kinases (MAPKs) kinase. In the presence of the TLR4 co-receptor CD14, LPS sensing further promotes TLR4 internalisation into endosomes, where it recruits another signalling adaptor, TIR-domain-containing adaptor protein inducing interferon-β (TRIF), to initiate type I interferon response, NF-κB and MAPKs signalling [3, 4].

To establish an infection, pathogenic microorganisms have evolved multiple mechanisms to subvert host immunity [5]. For example, pathogenic *Yersinia* that cause diseases ranging from plague to gastroenteritis, use a sophisticated bacterial type 3 secretion system to inject YopJ, a bacterial acetyltransferase into mammalian cells to inhibit the central signalling kinase (TGF-beta-activated kinase 1) TAK1 and dismantle downstream NF-κB and MAPKs activation [6]. Although this effectively suppresses proinflammatory gene expression, blockade of TAK1 and its downstream kinases such as inhibitor of nuclear factor kappa-B kinase subunit (IKK)α/β and (MAPKAPK2) MK2 prevents inhibitory phosphorylation of receptor-interacting protein kinase 1 (RIPK1) that is anchored on Complex I or the TLR4-TRIF complex [7–9]. As such, RIPK1 undergoes activating autophosphorylation and assembles a secondary death-inducing complex comprising the core components RIPK1, FAS-associated death domain protein (FADD) and caspase-8 to trigger cell death and protect against *Y. pseudotuberculosis* infection [9–13]. To discriminate the death-inducing complexes downstream of TNFR1 and TLR4-TRIF, these signalling platforms were dubbed ‘Complex IIb’ [1, 14] and ‘TRIFosome’ [15], respectively. Building on these studies, we and others recently identified two additional substrates, the pore-forming proteins gasdermin D (GSDMD) and GSDME, that are activated in different cell types downstream of caspase-8 that is crucial in driving anti-*Yersinia* defence [16–19]. In macrophages, caspase-8 directly cleaves GSDMD to release its cytotoxic N-terminal fragment to trigger pyroptosis [17–19], while in neutrophils, activation of downstream caspase-3 promotes GSDME cleavage, neutrophil pyroptosis and IL-1β release [16]. Together, these observations highlight that RIPK1-dependent death-inducing complex activates distinct gasdermins in neutrophils and macrophages and led us to examine whether the upstream signalling requirement to trigger RIPK1 activation is distinctly regulated in these two cell types.

Here, we report that, unlike macrophages that readily undergo RIPK1-dependent pyroptosis during *Y. pseudotuberculosis* infection, neutrophils minimally require IFN-γ priming to trigger RIPK1-dependent caspase-8 activation, while IFN-γ and LPS co-priming triggers maximal lysis and cytokine secretion. In addition, while both TNFR1 and TRIF signalling can drive RIPK1-dependent pyroptosis in macrophages, neutrophils exclusively signal via TNFR1 to drive RIPK1-dependent pyroptosis. Lastly, our study demonstrates that autocrine TNFR1 signalling is critical for pro-IL-1β expression in neutrophils, while cytokine priming occurs via TRIF signalling in macrophages. Overall, our study highlights the emerging theme of cell type-specific innate and death signalling in closely related cells.

## Results

### IFN-γ priming is required to trigger RIPK1-dependent pyroptosis in neutrophils during *Y. pseudotuberculosis* infection in vitro

**Fig. 1:**
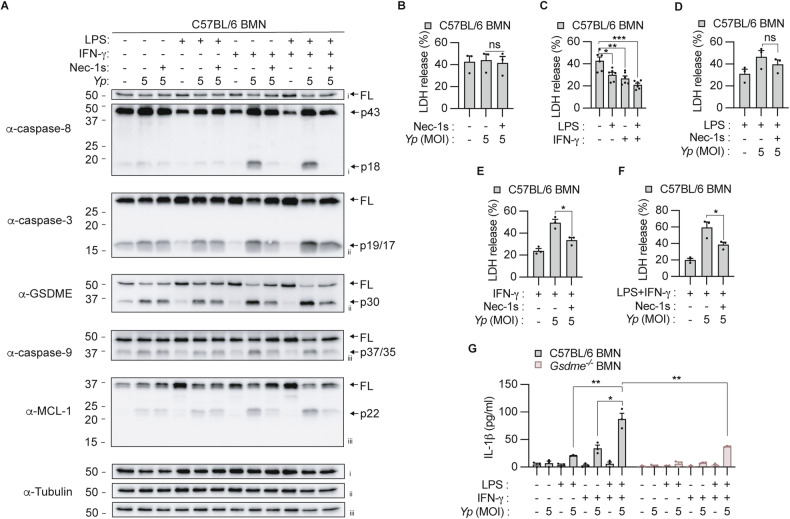
IFN-γ priming is required to trigger RIPK1-dependent caspase-8 activation in neutrophils following *Y. pseudotuberculosis* infection in vitro. **A**–**G** Bone marrow neutrophils (BMNs) were left unstimulated or primed with 100 ng/ml LPS, 100 ng/ml IFN-γ or both LPS and IFN-γ for 3 h prior to infection with *Y. pseudotuberculosis* (*Yp*) at a multiplicity of infection(MOI) of 5 for 4 h. Where indicated, cells were treated with 50 μM Necrostatin-1s (Nec-1s) 20–30 min before infection. **A** Mixed supernatant and cell extracts were examined by immunoblotting. Three membranes were used for immunoblotting (i, ii, iii). **B**–**F** LDH release and **G** IL-1β secretion were measured at the end of the infection. **B**–**G** Data are represented as mean + SEM cell stimulation from at least three independent experiments. *P < 0.05, **P < 0.01 and ***P < 0.001.

We recently demonstrated that *Y. pseudotuberculosis* infection triggers RIPK1-dependent caspase-8 activation and downstream GSDME-dependent pyroptosis and IL-1β release in neutrophils [16]. In these experiments, we primed neutrophils with LPS to induce expression of the caspase-1/8 substrate, pro-IL-1β, and co-stimulated these cells with IFN-γ to promote the expression of anti-apoptotic proteins to limit spontaneous neutrophil apoptosis [20]. However, it is unclear whether simultaneous LPS and IFN-γ priming is an absolute requirement to elicit RIPK1-dependent neutrophil pyroptosis during *Y. pseudotuberculosis* infection. To examine this, we infected neutrophils with *Y. pseudotuberculosis* in the presence and absence of priming. In agreement with our previous study [16], approximately 40% of unprimed neutrophils released lactate dehydrogenase (LDH) in a GSDME-dependent manner after 7 h in culture (Supplementary Fig. 1A), and displayed spontaneous caspase-8, -9 and -3 activation and resultant GSDME cleavage (Fig. 1A). In unprimed neutrophils*, Y. pseudotuberculosis* infection induced caspase-9, caspase-3 and GSDME processing, but not caspase-8 cleavage compared to uninfected cells (Fig. 1A). Although *Y. pseudotuberculosis* infection induced GSDME cleavage in unprimed cells, this increase was not sufficient to further promote neutrophil lysis compared to uninfected neutrophils undergoing spontaneous apoptosis (Fig. 1B). To discriminate RIPK1 kinase-dependent caspase-8 activation from spontaneous apoptosis, we treated neutrophils with a RIPK1 kinase inhibitor, Nec-1s, before *Y. pseudotuberculosis* infection. Nec-1s treatment did not reduce *Y. pseudotuberculosis-*induced lysis, apoptotic caspase processing, and GSDME cleavage in unprimed neutrophils (Fig. 1A, B). Together, these data suggest that *Y. pseudotuberculosis* infection promotes intrinsic apoptosis but not RIPK1-dependent caspase-8 activation in unprimed neutrophils.

LPS priming induced expression of the anti-apoptotic protein, MCL-1, resulting in reduced spontaneous caspase-9, -3, GSDME processing, and neutrophil lysis (Fig. 1A, C; Supplementary Fig. 1B–D). *Y. pseudotuberculosis* infection induced caspase-9, caspase-3 and GSDME processing in LPS-primed neutrophils (Fig. 1A), indicating that LPS priming was insufficient to restrict *Y. pseudotuberculosis*-induced intrinsic apoptosis. However, Nec-1s treatment did not suppress neutrophil lysis (Fig. 1D) and processing of apoptotic caspases and GSDME (Fig. 1A). These indicate that like unprimed neutrophils (Fig. 1A, B), LPS priming does not sensitize neutrophils to RIPK1-dependent caspase-8 activation upon *Y. pseudotuberculosis* infection. Consistent with LPS priming, IFN-γ priming and LPS-IFN-γ co-priming also promoted MCL-1 expression, reduced apoptotic caspase activation and spontaneous neutrophil lysis (Fig. 1A, C, Supplementary Fig. 1B–D). Under such conditions, caspase-8 processing and cleavage of downstream substrates including caspase-3, MCL-1, GSDME, and LDH release were all sensitive to Nec-1s inhibition upon *Y. pseudotuberculosis* infection (Fig. 1A, E, F). Taken together, these results indicate that IFN-γ priming alone is sufficient to trigger RIPK1-dependent caspase-8-activation following *Y. pseudotuberculosis* infection in neutrophils (Fig. 1A, E), while LPS/IFN-γ co-priming enables maximal cell lysis and IL-1β release (Fig. 1F, G).

### TNFR1 but not TRIF signalling is required for both RIPK1-mediated pyroptosis, IL-1α and IL-1β production in neutrophils

**Fig. 2:**
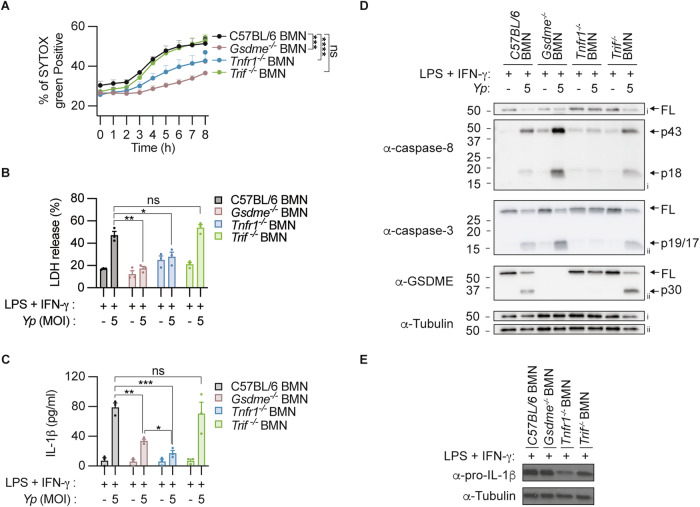
TNFR1 but not TRIF signalling is required for RIPK1-dependent caspase-8-mediated death and IL-1β production in neutrophils. **A**–**D** BMNs were primed with 100 ng/ml LPS and 100 ng/ml IFN-γ for 3 h and infected for 4 h with *Y. pseudotuberculosis* (*Yp*) at a multiplicity of infection (MOI) of 5 for 4 h. **A** Percentage of SYTOX Green uptake **B** LDH release, **C** IL-1β secretion and **D** immunoblots from mixed supernatant and cell extracts were examined at 4 h post-infection. Two membranes were used for immunoblotting (i, ii). **E** Pro-IL-1β levels from uninfected BMNs were examined after LPS and IFN-γ co-priming for 7 h. **A**–**C** Data are represented as mean + SEM cell stimulation from three independent experiments. *P < 0.05, **P < 0.01, ***P < 0.001 and ns *P* > 0.05.

Previous studies reported that both TNFR1 and TLR4-TRIF signalling contribute to RIPK1-dependent caspase-8 activation during *Y. pseudotuberculosis* infection in macrophages [11, 15, 18, 19]. However, the upstream signalling requirements for RIPK1-dependent caspase-8 activation in neutrophils during *Y. pseudotuberculosis* infection remain unclear. Since LPS and IFN-γ co-priming result in robust neutrophil pyroptosis and IL-1β release during *Y. pseudotuberculosis* infection (Fig. 1A, F, G), we applied this priming regimen and compared the response of *Tnfr1*^−^^*/*^^−^ and *Trif*^−^^*/*^^−^ neutrophils to wild-type and *Gsdme*^−^^*/*^^−^ neutrophils following *Y. pseudotuberculosis* infection. As expected and consistent with our previous report [16], *Y. pseudotuberculosis* triggered neutrophil pyroptosis and IL-1β release in a GSDME-dependent manner, as uptake of the membrane-impermeable dye SYTOX Green (Fig. 2A), LDH release (Fig. 2B) and IL-1β release (Fig. 2C) were significantly reduced in *Gsdme*^−^^*/*^^−^ compared to wild-type neutrophils. To our surprise, unlike in macrophages [11, 15], *Trif*^−^^*/*^^−^ neutrophils displayed robust caspase-8, caspase-3 and GSDME cleavage, and indistinguishable SYTOX Green uptake, LDH release and IL-1β secretion compared to wild-type neutrophils following *Y. pseudotuberculosis* infection (Fig. 2A–D). In contrast, *Tnfr1* deficiency significantly reduced neutrophil lysis upon *Y. pseudotuberculosis* infection compared to wild-type neutrophils in IFN-γ or LPS/IFN-γ-primed cells (Fig. 2A, B; Supplementary Fig. 2A). *Tnfr1*^−^^*/*^^−^ neutrophils also displayed impaired caspase-8, caspase-3 and GSDME activation compared to wild-type and *Trif*^−^^*/*^^−^ neutrophils (Fig. 2D). Interestingly, *Tnfr1* deficiency further reduced IL-1β release compared to *Gsdme*^−^^*/*^^−^ neutrophils (Fig. 2C), while soluble TNF release remain comparable between all genotypes (Supplementary Fig. 2B). Subsequent immunoblot analysis revealed impaired pro-IL-1β expression in *Tnfr1*^−^^*/*^^−^ compared to wild-type neutrophils (Fig. 2D), indicating that autocrine TNFR1 signalling promotes pro-IL-1β expression in neutrophils, consistent with a recent report [21]. Notably, *Gsdme*^−^^*/*^^−^ neutrophils displayed more caspase-8 and -3 processing compared to wild-type and *Trif*^−^^*/*^^−^ neutrophils following *Y. pseudotuberculosis* infection (Fig. 2D), likely due to sustained caspase processing in the absence of cell lysis, as previously reported in inflammasome-activated *Gsdmd*^−^^*/*^^−^ macrophages [22, 23]. Together, these data indicate that LPS/IFN-γ co-priming sensitizes neutrophils to TNFR1-driven RIPK1-dependent pyroptosis, pro-IL-1β expression and mature IL-1β release during *Y. pseudotuberculosis* infection, while TRIF signalling is dispensable.

### IFN-γ promotes surface TNFR1 expression and enhances TNF secretion in neutrophils

**Fig. 3:**
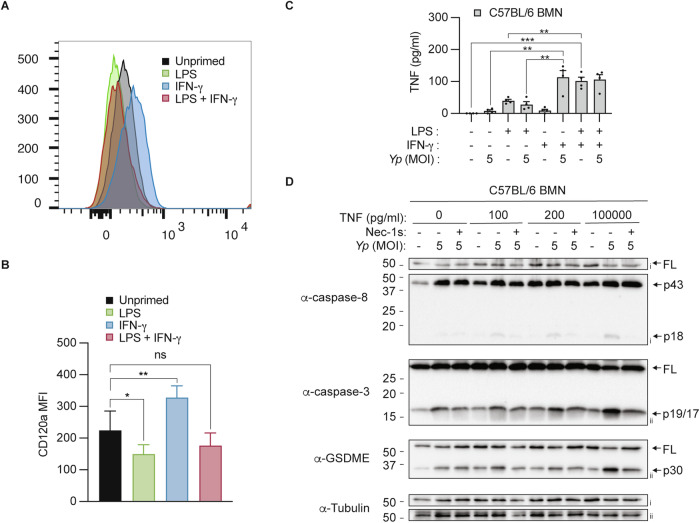
IFN-γ priming upregulates neutrophil TNFR1 expression and enhances TNF secretion during infection. **A**, **B** BMNs were left unprimed or primed with either 100 ng/ml LPS, 100 ng/ml IFN-γ or both LPS and IFN-γ for 3 h and the expression of TNFR1 (CD120a) was determined by flow cytometry. **A** Representative histogram of CD120a expression in unprimed and primed BMNs. **B** Mean fluorescent staining (MFI) of CD120a in unprimed and primed BMNs. **C** Bone marrow neutrophils (BMNs) were left unstimulated or primed with 100 ng/ml LPS, 100 ng/ml IFN-γ or both LPS and IFN-γ for 3 h before infection with *Y. pseudotuberculosis* (*Yp*) at a multiplicity of infection (MOI) of 5 for 4 h. TNF secretion was quantified at the end of infection. **D** BMNs were primed with increasing dose of recombinant TNF for 4 h before *Y. pseudotuberculosis* infection in the presence or absence of 50 μM Nec-1s. Immunoblots from mixed supernatant and cell extracts were examined at 4 h post-infection. Two membranes were used for immunoblotting (i, ii). Data are represented as mean + SEM cell stimulation from **B** three or **C** four independent experiments. *P < 0.05 and **P < 0.01.

Next, we sought to unravel the mechanisms by which IFN-γ and LPS/IFN-γ co-priming enables neutrophils to signal via TNFR1 to trigger RIPK1-dependent caspase-8 activation and downstream GSDME-mediated pyroptosis. We first performed flow cytometry to examine surface TNFR1 with and without priming. IFN-γ priming triggered a significant increase in surface TNFR1 expression compared to naïve neutrophils (Fig. 3A, B) and sensitized neutrophils to TNFR1-dependent cell death (Supplementary Fig. 2A). In contrast, LPS priming reduced neutrophil TNFR1 expression compared to naïve cells (Fig. 3A, B), consistent with the lack of RIPK1-driven pyroptosis following *Y. pseudotuberculosis* infection (Fig. 1A, D). Unexpectedly, while LPS-IFN-γ co-priming enables robust neutrophil pyroptosis and IL-1β release from *Y. pseudotuberculosis-*infected neutrophils (Fig. 1F, G), this priming regimen did not induce TNFR1 expression compared to naïve neutrophils (Fig. 3A, B). Thus, we next examined soluble TNF release from neutrophils in the presence and absence of priming and infection. Consistent with a lack of RIPK1-dependent pyroptosis (Fig. 1C, D), soluble TNF was barely detected in naïve or LPS-primed neutrophils, regardless of *Y. pseudotuberculosis* infection (Fig. 3C). Although IFN-γ priming alone did not induce TNF release, IFN-γ-primed neutrophils released 5-fold more TNF compared to unprimed or LPS-primed neutrophils following *Y. pseudotuberculosis* infection (Fig. 3C). LPS/IFN-γ co-priming by itself triggered similar levels of soluble TNF release compared to IFN-γ-stimulated neutrophils that were exposed to *Y. pseudotuberculosis* (Fig. 3C). This suggests that LPS/IFN-γ co-priming induced sufficient levels of soluble TNF that sensitises neutrophils to RIPK1-dependent caspase-8 activation following *Y. pseudotuberculosis* infection. Since LPS/IFN-γ co-priming triggered 100-200 pg/ml of soluble TNF release (Fig. 3C), we reasoned that supplementing neutrophils with exogenous recombinant TNF at this dosage would be sufficient to trigger RIPK1-dependent caspase-8 activation. To verify this hypothesis, we titrated increasing doses of recombinant soluble TNF in naïve neutrophils and infected these cells with *Y. pseudotuberculosis*. Indeed, we observed that 100-200 pg/ml of recombinant TNF is sufficient to promote RIPK1-dependent caspase-8 activation following *Y. pseudotuberculosis* infection in a dose-dependent manner (Fig. 3D). Since TNF priming does not suppress basal neutrophil apoptosis (Supplementary Fig. 3), these cells still display spontaneous caspase-3 and GSDME cleavage (Fig. 3D). Collectively, our data demonstrate that IFN-γ priming upregulates TNFR1 expression and sensitize neutrophils to release robust TNF upon infection, while LPS-IFN-γ co-priming promotes RIPK1-dependent pyroptosis by producing sufficient soluble TNF prior to infection.

### TRIF signalling primes macrophage cytokine production while autocrine TNF is dispensable

**Fig. 4:**
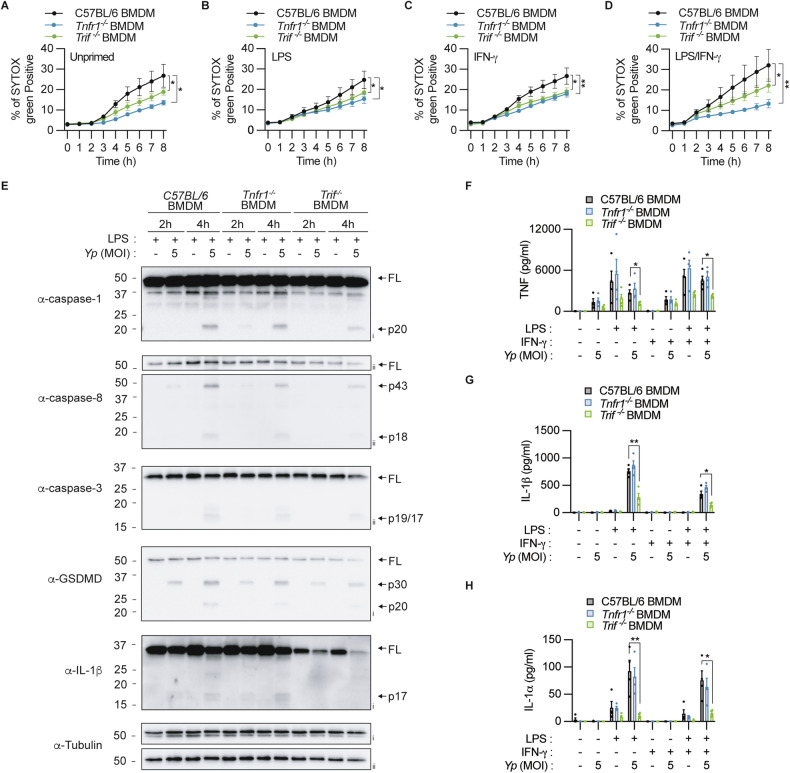
TNFR1 and TRIF signalling are required for macrophage RIPK1-driven pyroptosis, but only TRIF signalling affects cytokine production in macrophages. BMDMs were left **A** unstimulated or primed with **B** 100 ng/ml LPS, **C** 100 ng/ml IFN-γ or **D** both LPS and IFN-γ for 3 h before infecting with *Y. pseudotuberculosis* (*Yp*) (MOI 5). **A**–**D** Percentage SYTOX Green uptake was quantified over 8 h. **E** BMDMs were primed 100 ng/ml LPS for 3 h and infected with *Y. pseudotuberculosis* (*Yp*) (MOI 5). Mixed supernatant and cell extracts were examined by immunoblotting at the indicated time points. Two membranes were used for immunoblotting (i, ii). **F**–**H** BMDMs were left unstimulated or primed with 100 ng/ml LPS, 100 ng/ml IFN-γ or both LPS and IFN-γ for 3 h before infecting with *Y. pseudotuberculosis* (*Yp*) (MOI 5) for 4 h. **F** TNF, **G** IL-1β and **H** IL-1α in the cell culture supernatant were quantified by ELISA. **A**–**D**, **F**–**H** Data are represented as mean + SEM cell stimulation from three independent experiments. *P < 0.05, **P < 0.01.

Our data above demonstrates that neutrophils minimally require IFN-γ priming to trigger RIPK1-dependent caspase-8 activation during *Y. pseudotuberculosis* infection. Although previous studies reported that macrophages utilize both TNFR1 and TLR4-TRIF axis to activate RIPK1-caspase-8 following *Y. pseudotuberculosis* infection in unprimed cells [11], it is unclear whether LPS, IFN-γ or LPS/IFN-γ co-priming triggers a bias to a specific signalling axis. To examine this, we applied the same priming and infection regimen as described above in neutrophils to macrophages and quantified cellular toxicity by SYTOX Green uptake. *Tnfr1* and *Trif* deficiency uniformly reduced SYTOX Green uptake (Fig. 4A–D) and caspase-8 processing (Fig. 4E, Supplementary Fig 4A–C) compared to wild-type macrophages following *Y. pseudotuberculosis* infection in all priming conditions examined, suggesting that *Y. pseudotuberculosis* infection triggers TNFR1, TRIF, and RIPK1-dependent lysis irrespective of priming.

Having established that, unlike neutrophils, macrophages utilize both TNFR1 and TLR4-TRIF to trigger RIPK1-dependent cell death during *Y. pseudotuberculosis* infection, we next examined the contribution of these receptors to cytokine production. In stark contrast to neutrophils, we observed that production of the proinflammatory cytokines, TNF, IL-1β and IL-1α all required TRIF signalling, while autocrine TNF appears dispensable (Fig. 4F–H). Although *Tnfr1* and *Trif* deficiency reduced SYTOX Green uptake, caspase-8, -3 and GSDMD processing, only *Trif* deficiency reduced caspase-1 activation following *Y. pseudotuberculosis* infection compared to wild-type cells (Fig. 4E, Supplementary Fig. 4C), which further impaired pro-IL-1β processing into the mature p17 fragment (Fig. 4E). Overall, our data indicate that macrophages do not require priming to trigger RIPK1-dependent pyroptosis but requires LPS-TRIF signalling to induce the expression and activation of IL-1β.

### TNFR1 and TRIF signalling promotes anti-*Yersinia* defence in vivo

**Fig. 5:**
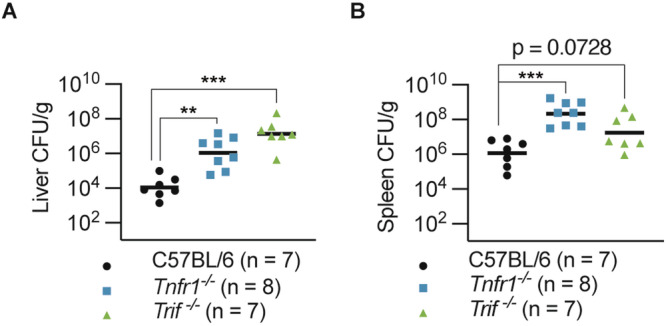
TNFR1 and TRIF promotes anti-*Yersinia* defence in vivo. **A**, **B** Mice were challenged orally with 2 × 10^8^ CFU *Y. pseudotuberculosis*. Bacterial load in the **A** liver and **B** spleen were quantified at 4 d post-infection. **A**, **B** Data are geometric means and pooled from two independent experiments. **P < 0.01, ***P < 0.001.

Next, we examined the contribution of TNFR1 and TRIF to combat *Y. pseudotuberculosis* infection in vivo. For this, we orally challenged wild-type, *Tnfr1*^−^^*/*^^−^ and *Trif*^−^^*/*^^−^ mice with *Y. pseudotuberculosis* and compared bacterial burden in these animals. Consistent with previous reports [11, 21], we recovered significantly more bacteria from the liver and spleen of *Tnfr1*^−^^*/*^^−^ mice compared to wild-type animals (Fig. 5A, B). Interestingly, while *Trif* deficiency impaired bacterial clearance in the liver compared to wild-type animals (Fig. 5A), splenic bacterial load was not statistically different between wild-type and *Trif*^−^^*/*^^−^ animals (Fig. 5B). Together, these data suggest that TNFR1 and TRIF signalling promote anti-*Yersinia* defence in different tissues.

## Discussion

RIPK1 kinase-dependent cell death is a potent inflammatory pathway that promotes host defence and inflammatory disease [10, 24, 25]. We previously demonstrated that neutrophils and macrophages utilize distinct pyroptotic effectors to counteract suppression of TAK1/IKK signalling by *Y. pseudotuberculosis* [16], consistent with the emerging theme that innate immune and cell death signalling are distinctly regulated in the myeloid compartment [26–28]. In this study, we uncovered additional cell type-specific requirements for RIPK1-dependent pyroptosis in myeloid cells. Unlike macrophages that readily undergo RIPK1-dependent pyroptosis following *Y. pseudotuberculosis* infection, we report that neutrophils minimally require IFN-γ or TNF priming to activate RIPK1-dependent pyroptosis; while IFN-γ and LPS co-priming licenses maximal neutrophil pyroptosis and IL-1β release. A previous study reported that *Y. pseudotuberculosis* suppresses IFN-γ production from human and murine γδ T cells [29]. However, it is unclear how suppression of IFN-γ, a signature Th1 cytokine that is best known to promote macrophage clearance of intracellular pathogens, is beneficial for *Y. pseudotuberculosis* since it largely exhibits an extracellular lifestyle. Although IFN-γ is likely to exert pleiotropic effects on the various cell types, our data suggest that neutrophils are likely a key target of IFN-γ during *Y. pseudotuberculosis* infection, possibly by licensing RIPK1 activation, GSDME-dependent pyroptosis and secretion of the proinflammatory cytokines IL-1β and TNF. While programmed neutrophil lysis such as pyroptosis and NETosis are emerging as key anti-microbial defence mechanisms, dysregulation in neutrophil lysis is increasingly associated with diseases. For instance, systemic lupus erythematosus (SLE) is an autoimmune disease that is characterised by high levels of autoantibodies against proteinase-3 (PR3) and myeloperoxidase (MPO), proteins that are highly expressed and sequestered within neutrophil granules. This has led to a model where neutrophil rupture via NETosis releases MPO and PR3 to drive the pathogenesis of SLE [30]. However, several studies have refuted this possibility in mice, since animals that are genetically deficient in activating NETosis are not protected from experimental SLE [31–33]. In contrast, a recent study reported that *Gsdme*-deficient animals are significantly protected from experimental SLE compared to wild-type animals [34], putting forward GSDME as a prime candidate that mediates neutrophil lysis and ensuing MPO and PR3 release. Since TNF and IFN-γ signalling are required for the pathogenesis of SLE [35, 36], it is tempting to speculate that IFN-γ signalling licenses neutrophils to trigger GSDME-dependent pyroptosis and drive SLE. Future studies utilising neutrophil-restricted *Ifngr* knockout animals will be useful to dissect the role of neutrophil IFN-γ signalling during infection and SLE.

TLR4 and TNFR1 ligation drive potent proinflammatory and prosurvival responses. However, coincident detection of TAK1/IKK inhibition, such as YopJ from pathogenic *Yersinia*, promotes the assembly of a cytosolic RIPK1 kinase-dependent pyroptosis-inducing complex in macrophages [10, 18, 19]. Surprisingly, despite being notoriously labelled as short-lived cells, we found that neutrophils selectively activate RIPK1-dependent pyroptosis downstream of TNFR1 signalling, while TRIF remains dispensable. Although the mechanisms by which neutrophils disarms TLR4-TRIF-induced cell death is not further investigated in this study, we speculate that low expression of the TLR4 co-receptor, CD14, which promotes TLR4 endocytosis and recruitment of TRIF to endosomes may account for the lack of TLR4-driven cell death in these cells [3, 4, 37]. This cell type-specific adaptation has likely evolved to benefit the host because the primary function of neutrophils is to phagocytose invading pathogens and cellular debris during infection, thus disarming TLR4-dependent cell death can prolong their lifespan upon encountering Gram-negative pathogens at the site of infection. However, since our study was performed using bone marrow neutrophils, we do not rule out the possibility that elicited neutrophils may induce CD14 expression during infection or are able to reprogramme themselves to undergo TLR4-driven cell death at the site of infection. Indeed, a recent study proposed that TRIF signalling in neutrophils can contribute to TNF-induced lethality in vivo, although the proposed function of TRIF in that study was to potentiate TNF-driven cell death [38].

In summary, our findings highlight that cell death and innate immune signalling are distinctly regulated in myeloid cells and these cell type-specific adaptations have likely evolved to ensure a fine balance between cellular survival and cell death during anti-microbial defence in vivo.

## Materials and methods

### Mice

All mice were maintained in specific pathogen-free animal facility and were handled in accordance with the protocols approved by the Institutional Animal Care and Use Committee (IACUC) of the National University of Singapore. C57BL/6 J (strain #000664), *Gsdme*^−^^*/*^^−^ (strain #032411)*, Trif*^−^^*/*^^−^ (strain #005037) and *Tnfr1*^−^^*/*^^−^ (strain #003242) were bought from the Jackson Laboratory and bred in dedicated pathogen-free facilities at the National University of Singapore. Mice of either sex were euthanized at various ages ranging from 7–12 weeks.

### Primary myeloid cell culture

Bone marrow-derived macrophages (BMDMs) were differentiated in Dulbecco’s modified Eagle medium (Gibco) supplemented with 20% L929 supernatant (as a source of M-CSF), 10 mM HEPES (Thermo Fisher Scientific), 10% heat-inactivated fetal calf serum (Gibco) and nonessential amino acids (Gibco) and stimulated on days 7–9 of differentiation. Mature neutrophils were purified from murine bone marrow (BMNs) using anti-Ly6G microbeads (Miltenyi Biotec) (>98% purity) as previously described [16, 26, 27]. In all experiments, neutrophils were seeded at a density of 4 × 10^5^ cells per well in 200 μl of Opti-MEM (Thermo Fisher Scientific) and stimulated on the day of purification. Macrophages were seeded at a density of 5 × 10^4^ cells per well in complete media a day prior to stimulation.

### Cell viability and cytokine secretion assays

Cell lysis was quantified by measuring the amount of LDH released into the cell culture supernatant using cytotoxicity detection kit (Roche, Sigma–Aldrich). Cell permeability was quantified by measuring SYTOX Green (0.5 μM; Invitrogen) uptake using a fluorescent plate reader (IncuCyte; Sartorius). The percentage of LDH release and SYTOX Green uptake was calculated relative to 100% cell lysis in the untreated control. TNF, IL-1α and IL-1β levels in cell-free supernatant were measured by enzyme-linked immunosorbent assay (ELISA) following manufacturers’ protocols (All R&D systems DuoSet).

### *Y. pseudotuberculosis* infection

Log-phase *Y. pseudotuberculosis* strain 32777 was prepared and used to infect BMDM or BMN at a multiplicity of infection (MOI) of 5 as previously described [16]. Where indicated, cells were primed with *E. coli* 055:B5 LPS (100 ng/mL; Invivogen) and/or IFN-γ (100 ng/mL; BioLegend) for 3 h prior to infection. For in vivo infection, mice were fasted for 16 h and challenged with 2 × 10^8^ CFU stationary-phase bacteria by oral gavage. To determine bacterial burden, mice were euthanized 4 d post-infection and tissues were harvested, homogenized in 2 or 5 mL of sterile phosphate-buffered saline, and serially diluted on Luria–Bertani (LB) agar supplemented with irgasan (2 μg/ml; Sigma–Aldrich).

### Immunoblotting

Cell culture supernatants were precipitated with methanol and chloroform using standard methods [39] and resuspended in cell extracts lysed in lysis buffer (66 mM Tris-Cl [pH 7.4], 10 mM DTT, 2% SDS (Sodium Dodecyl Sulphate), NuPage LDS sample buffer; Thermo Fisher Scientific). Proteins were boiled at 95 °C before being separated on 15% or 12% polyacrylamide gels and transferred onto nitrocellulose membrane using Transblot Turbo (Bio-Rad). Antibodies for immunoblot were against caspase-1 (AG-20B-0042-C100; 1:1000; Adipogen), full-length caspase-8 (D35G2; 1:1000; Cell Signalling Technology), cleaved caspase-8 (9429; 1:1000; Cell Signalling Technology), caspase-3 (9662 S; 1:1000; Cell Signalling Technology), GSDMD (EPR19828; 1:1000; Abcam), GSDME (EPR19859; 1:1000; Abcam), MCL-1 (D35A5; 1:1000; Cell Signalling Technology), IL-1β (AF-401-NA; 1:1000; R&D systems) and tubulin (DM1A; 1:5000; Abcam) (2144; 1:5000; Cell Signalling Technology). Nitrocellulose membranes were developed using Clarity (Bio-Rad) or SuperSignal West Femto (Thermo) and imaged using the ChemiDoc Imaging System (Bio-Rad) or X-ray film. Protein fold change was quantified through ImageJ using unprimed samples as the reference.

### Flow cytometry

Unprimed and primed BMNs were stained with fluorophore-conjugated anti-mouse antibodies against CD120a (55R-286; BioLegend) for TNFR1 expression. To examine neutrophil purity, purified mature neutrophils from the bone marrow were stained with α-Ly6G (1A8; BioLegend) to identify the neutrophil population. Cell profiles were acquired using BD LSRFortessa™ X-20 and analyzed using FlowJo (Tree Star). All samples were blocked with TruStain FcX (anti-mouse CD16/32; BioLegend) before staining.

### Statistical analyses

Statistical analyses were done using Prism 9 (GraphPad) software. Normality and Lognormality tests were first performed to determine the sample distribution. For samples that were normally distributed, unpaired *t*-test was performed but if the samples failed the normality test, the Mann–Whitney test was performed instead. A one-way ANOVA was used to analyze time course analysis for SYTOX Green uptake. For in vitro studies, data were expressed as mean values with SEM while geometric means were used to plot the data for in vivo studies. Where applicable, each dot represents a biological repeat. Data were considered significant when *p* ≤ 0.05 (∗), 0.005 (∗∗) or 0.001(∗∗∗).

### Supplementary information

Supplementary material

Uncropped gels

## Data Availability

All study data are included in the article and/or SI Appendix.
