# Supplementary material for: Differential signalling requirements for RIPK1-dependent pyroptosis in neutrophils and macrophages
Source: Cell Death Dis. 2024 Jul 4;15(7):479. doi: 10.1038/s41419-024-06871-8 (PMC11224406; doi:10.1038/s41419-024-06871-8)
Supplement: Supplementary file 1 — Supplementary material [file 41419_2024_6871_MOESM1_ESM.docx]

**Supplementary material**

**Differential signalling requirements for RIPK1-dependent pyroptosis in neutrophils and macrophages**

See Jie Yow^1,2^, Safwah Nasuha Rosli^1,2^, Paul E Hutchinson^2^, Kaiwen Chen^1,2,#^

^1^Immunology Translational Research Programme, Department of Microbiology and Immunology, Yong Loo Lin School of Medicine, National University of Singapore, Singapore.

^2^Immunology Programme, Life Sciences Institute, National University of Singapore, Singapore.

^#^Correspondance to: [kaiwen.chen@nus.edu.sg](mailto:kaiwen.chen@nus.edu.sg)


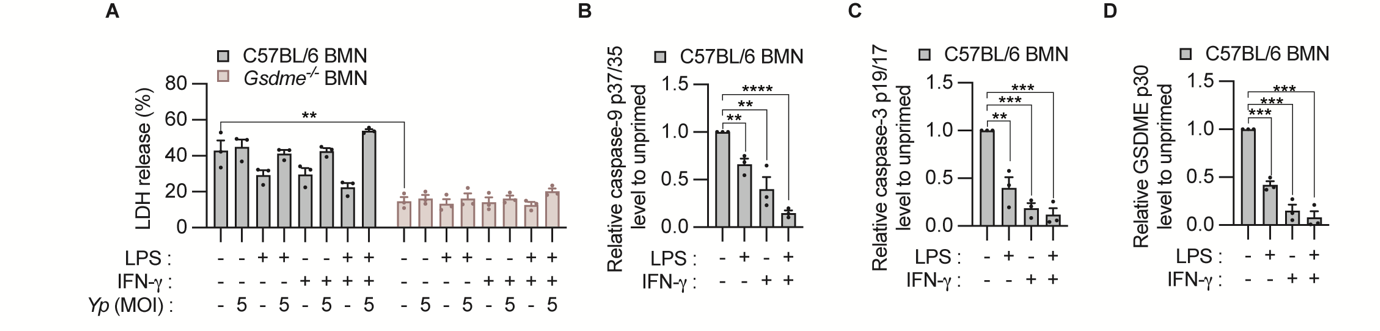


**Supplementary Figure 1. Unprimed neutrophils undergo spontaneous apoptotic caspase and GSDME activation. (A)** Bone marrow neutrophils (BMNs) were left unstimulated or primed with 100 ng/ml LPS, 100 ng/ml IFN-γ or both LPS and IFN-γ for 3h before infecting with *Y. pseudotuberculosis* (*Yp*) (MOI 5) for 4h. Percentage LDH release were quantified. **(B-D)** (BMNs) were left unstimulated or primed with 100 ng/ml LPS, 100 ng/ml IFN-γ or both LPS and IFN-γ for 7h. Relative amounts of cleaved **(B)** caspase-9, **(C)** caspase-3 and **(D)** GSDME cleavage against unstimulated neutrophils were quantified. Data are represented as mean + SEM cell stimulation from three independent experiments. *P < 0.05, **P < 0.01, ***P < 0.001, ****P < 0.0001.


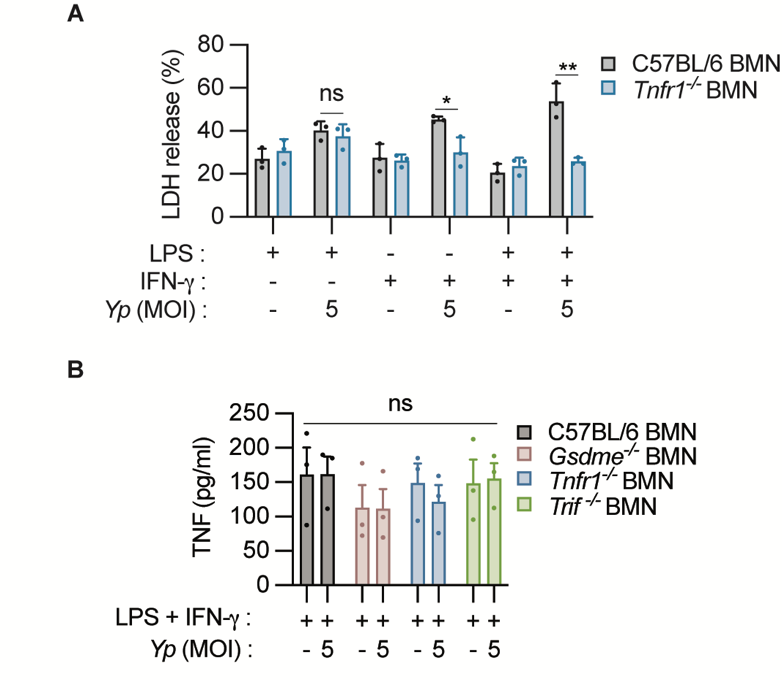


**Supplementary Figure 2. TNFR1 signalling promotes cell death in IFN-γ stimulated neutrophils upon *Y. pseudotuberculosis* infection.** **(A)** BMN were primed with 100 ng/ml LPS, 100 ng/ml IFN-γ or both LPS and IFN-γ for 3h prior to infection with *Y. pseudotuberculosis* (*Yp*) (MOI 5) for 4h. Percentage LDH release at 4h post-infection was quantified. **(B)** BMNs were primed with 100 ng/ml LPS and 100 ng/ml IFN-γ for 3h and infected for 4h with *Y. pseudotuberculosis* (*Yp*) at a multiplicity of 5 (MOI) for 4h. TNF secretion was quantified at the end of infection. Data are mean + SEM cell stimulation from three independent experiments. *P < 0.05, **P < 0.01.


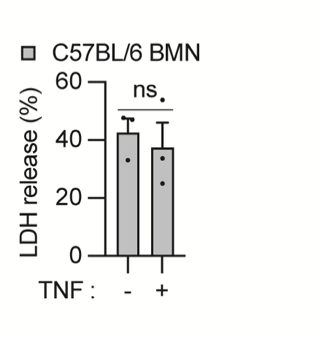


**Supplementary Figure 3. TNF priming is not sufficient to suppress spontaneous apoptosis in neutrophils.** BMDM left unstimulated or primed with 100 ng/ml recombinant TNF for 7h. Percentage LDH release in the cell culture supernatant was quantified. Data are represented as mean + SEM cell stimulation from three independent experiments.


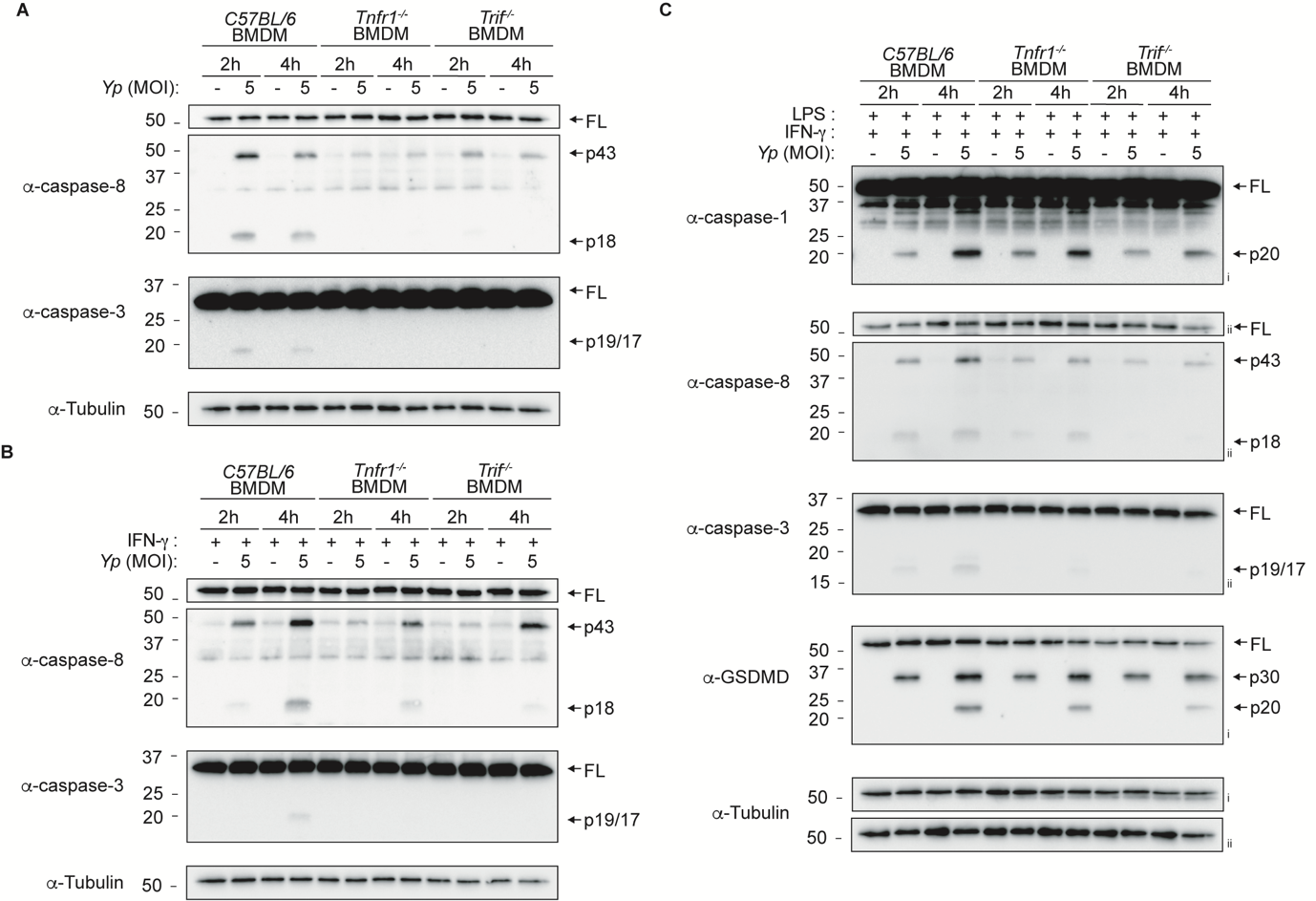


**Supplementary Figure 4. TNFR1 and TRIF collaborates to drive BMDM death during *Y. pseudotuberculosis* infection regardless of priming.** BMDMs were left **(A)** unstimulated or primed with **(B)** 100 ng/ml IFN-γ or **(C)** both LPS and IFN-γ for 3h before infecting with *Y. pseudotuberculosis* (*Yp*) (MOI 5). Where indicated, two membranes were used for immunoblotting (i, ii). Mixed supernatant and cell extracts were examined by immunoblotting at the indicated time points.
