# Supplementary material for: Differential signalling requirements for RIPK1-dependent pyroptosis in neutrophils and macrophages
Source: Cell Death Dis. 2024 Jul 4;15(7):479. doi: 10.1038/s41419-024-06871-8 (PMC11224406; doi:10.1038/s41419-024-06871-8)

Figure 1A uncropped western blots

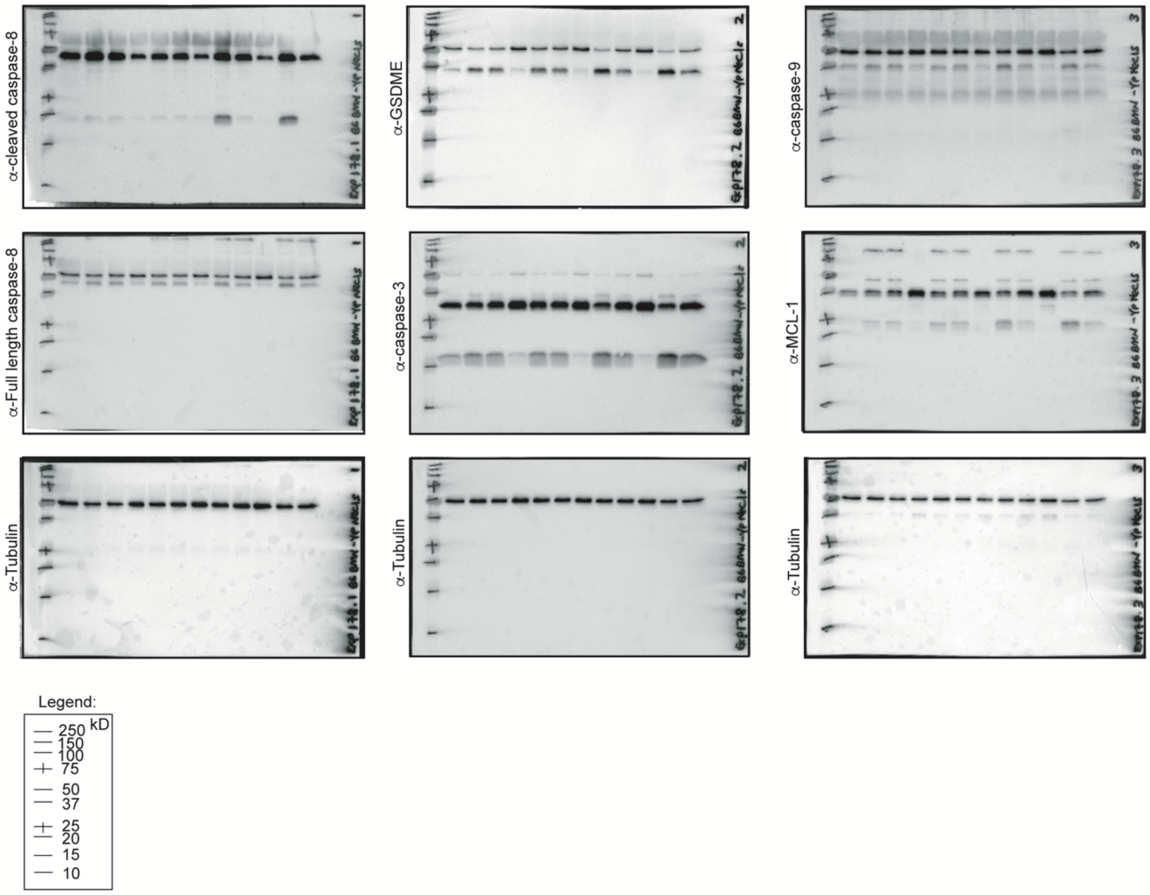

Figure 2D uncropped western blots

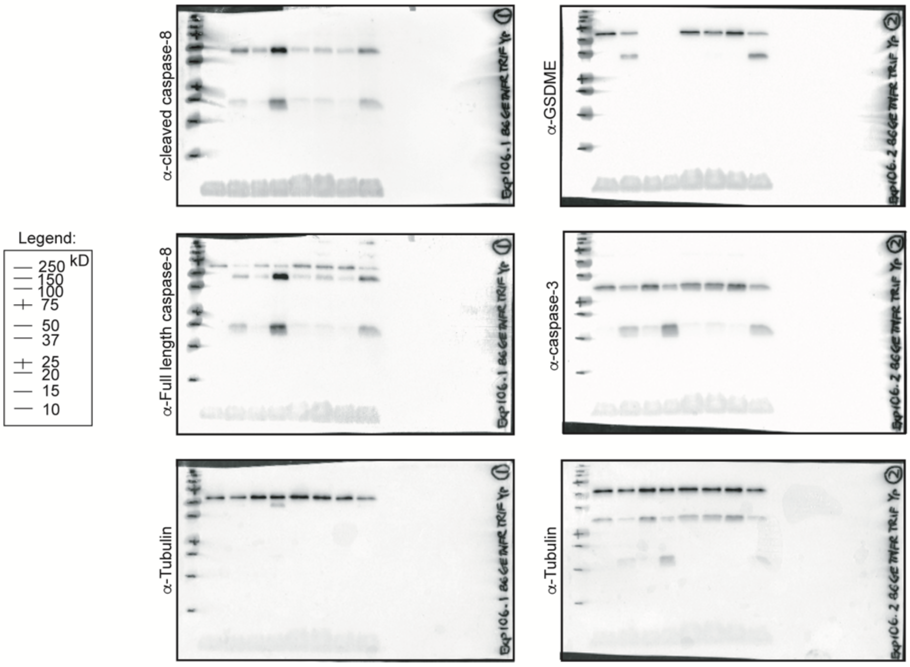

Figure 2E uncropped western blots

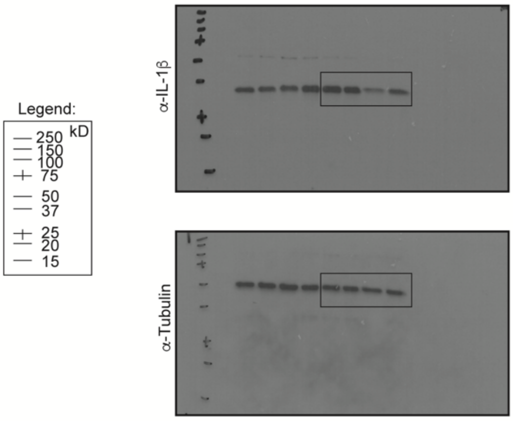

Figure 3D uncropped western blots

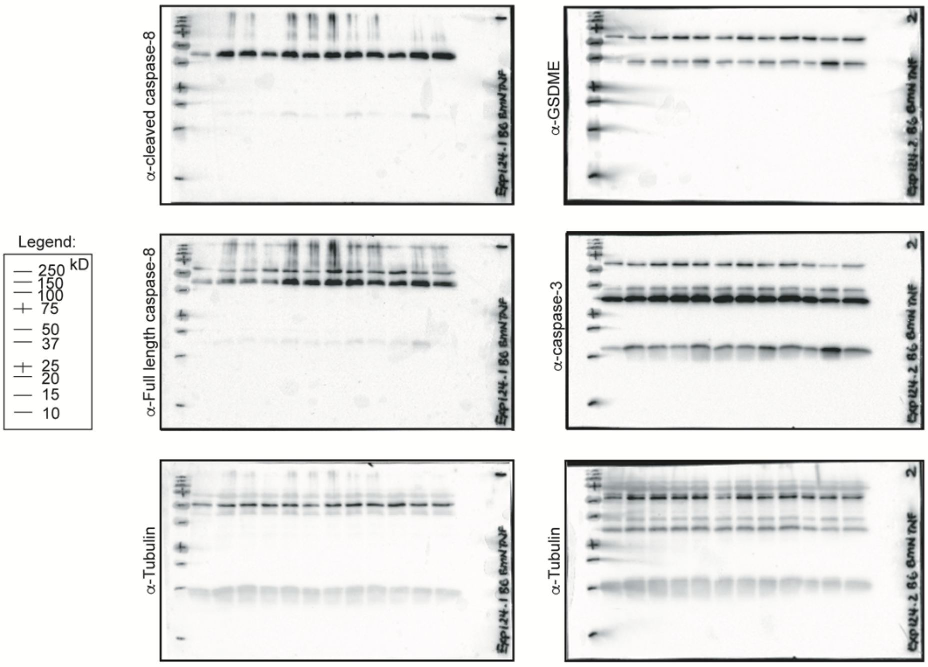

Figure 4E uncropped western blots

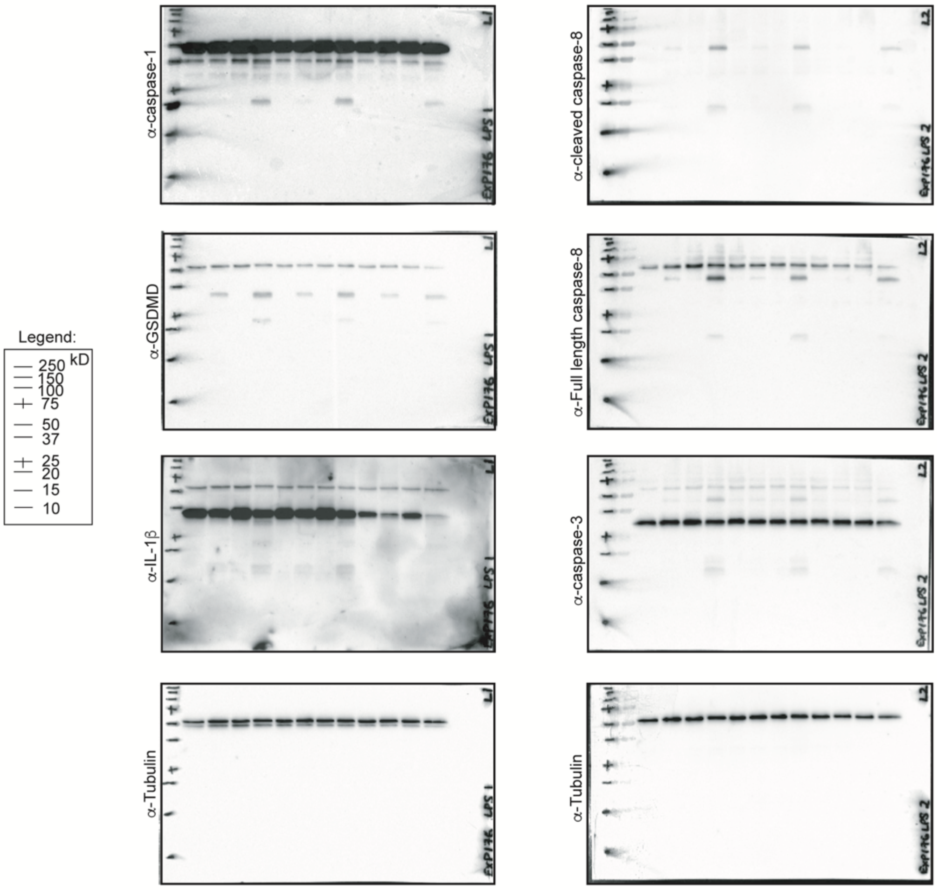

Supplementary figure 4A uncropped western blots

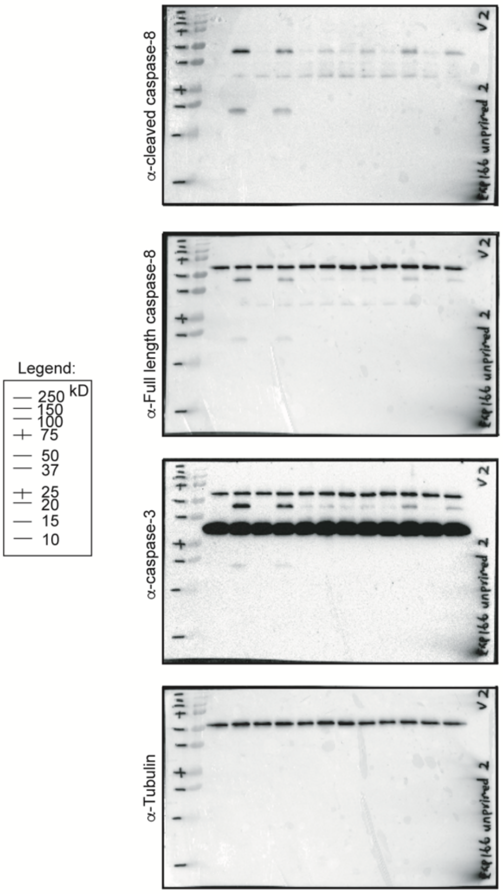

Supplementary figure 4C uncropped western blots

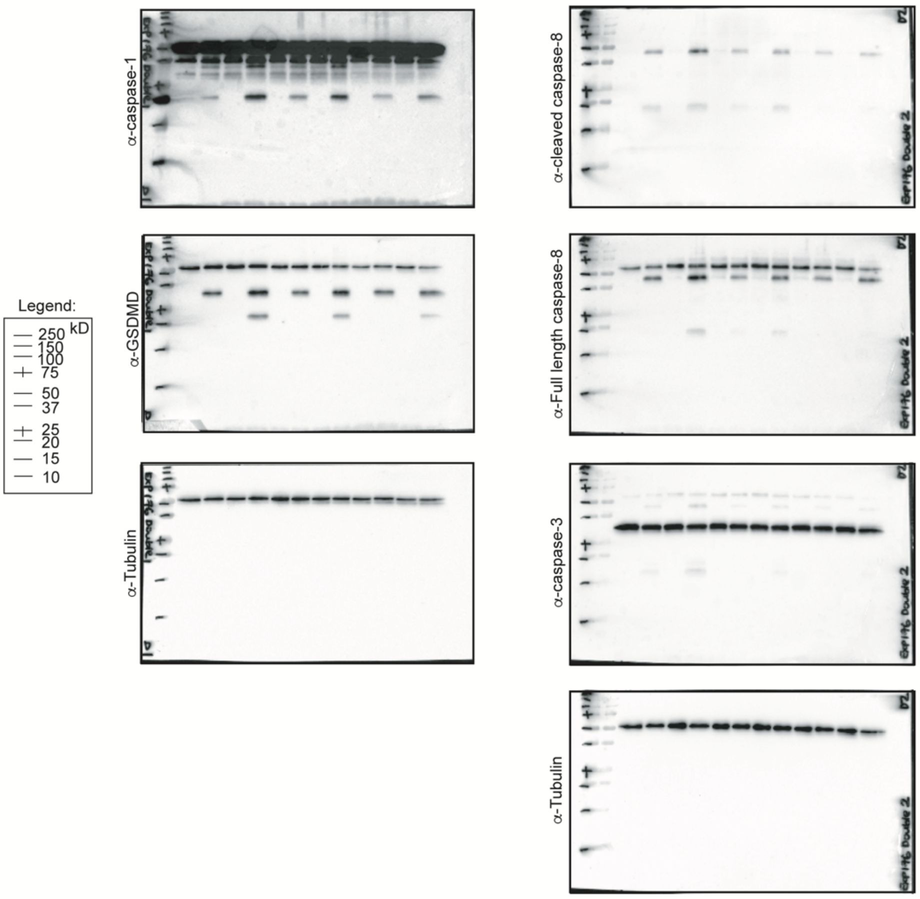

Supplement: Supplementary file 2 — Uncropped gels [file 41419_2024_6871_MOESM2_ESM.pdf]
